# Comment on: “Senolytic Treatment Improve Small Intestine Regeneration in Aging”

**DOI:** 10.14336/AD.2024.1262

**Published:** 2025-10-30

**Authors:** Hirofumi Rokutan

**Affiliations:** Department of Pathology & Research Team for Geriatric Pathology, Tokyo Metropolitan Institute for Geriatrics and Gerontology, Tokyo, Japan

**To the Editor,**

With great interest, I read the recent article by Luo et al. [1], who reported the beneficial effect of dasatinib and quercetin (D+Q) against age-related senescence in the small intestine. Although the study involved only a small sample, Luo et al. clearly demonstrated that the senolytic cocktail (D+Q) enhanced the clearance of apoptotic cells and the morphological recovery of the villi. I believe that their article will enable future studies to improve nutrient absorption in the elderly and even to develop new strategies for patients with Crohn’s disease, which can be associated with senescent cells in the intestine [2].

In addition to the effect of D+Q, Luo et al. also demonstrated a decrease in the number of Ki67-positive cells in old mice compared to young mice. This data, which is compatible with the villus/crypt structure profiles, is also important because previous results regarding the effect of aging on cell proliferation in the small intestine were contradictory [3, 4].

I just have two questions. First, did the beneficial effect of D+Q on old mice accelerate during treatment? A positive spiral via increased drug absorption might occur, when taking into consideration the regenerated and preserved small intestine epithelium, which is clearly shown morphologically in their model and also supported by their GSEA analysis. Second, why did the p16 expression not sufficiently reduce in one old mouse even after D+Q treatment (Fig. 1B, the rightmost band showing p16 expression)? Is it due to the incomplete sensitivity of the cellular senescence marker (due to the heterogeneous nature of senescent cells [5]), rather than the individual variability of the effect of D+Q among mice?
